# Evaluation of the prescription amount of narcotic analgesics based on cancer pain relief management

**DOI:** 10.1093/eurpub/ckac131.281

**Published:** 2022-10-25

**Authors:** J Park

**Affiliations:** Department of Pharmaceutical Sciences, International University of Health and Welfare, Fukuoka, Japan

## Abstract

**Background:**

A basic plan for promoting pain relief management for cancer patients was formulated in Japan, and proper use of narcotic analgesics is being promoted to relieve cancer pain. We aimed to explore the relationship between age and opioid narcotic dose in cancer patients and examine the effects of healthcare systems and social policies to prevent opioid abuse and addiction in a national cohort in Japan.

**Methods:**

Data were collected from April 2014 to March 2019 from the nationwide health insurance claims open database in Japan. Prescription data were collected for opium alkaloids and synthetic narcotics. A multivariate logistic regression analysis was performed based on the age groups and in- or out-of-hospital prescription quantities (mg), taking into account equivalences in quantities of opioids prescribed yearly on the hypothesized relationships between variables.

**Results:**

The average annual quantities of opium alkaloid prescribed gradually decreased by 2.8% (mg = 10,249,115) over six years, especially in in-hospital prescriptions. On the other hand, in-hospital annual prescriptions of synthetic narcotics increased by 24.1% (mg = 2,135,568), out-of-hospital opium alkaloid by 1.3% (mg = 37,565,229), and synthetic narcotics by 25.9% (mg = 1,404,641). Among the demographic variables, cancer patients aged 65 years or older were significantly associated with the types of opioid variables (p < .001) and annual reductions in prescribed amounts (p < .001).

**Conclusions:**

Treatment with opium alkaloids and synthetic narcotics increased among outpatients in the years examined; however, the trend varied across age groups. Our findings suggest that improvements in early detection and treatment have resulted in a rise in the number of cancer patient survivors, thereby leading to an increase in narcotic analgesic dose. The palliative care for cancer patients will contribute as an epidemiological indicator to review the evidence-based health-related impact on public health.

**Key messages:**

• Treatments other than medical intervention should also be considered, taking into account the increase in the number of outpatient opioid prescription recipients due to increased cancer survival.

• Social interventions to prevent opioid abuse during cancer pain relief will contribute to preventing substance abuse by promoting collaboration between clinicians and local communities.

